# A Case-Control Study Brings to Light the Causes of Screen Failures in Phase 1 Cancer Clinical Trials

**DOI:** 10.1371/journal.pone.0154895

**Published:** 2016-05-05

**Authors:** Emmanuelle Kempf, Nathalie Lemoine, Gabrielle Tergemina-Clain, Anthony Turpin, Sophie Postel-Vinay, Emilie Lanoy, Jean-Charles Soria, Christophe Massard, Antoine Hollebecque

**Affiliations:** 1 Gustave-Roussy Cancer Campus, Drug Development Department, Villejuif, France; 2 Gustave-Roussy Cancer Campus, Biostatistics and Epidemiology unit, Villejuif, France; 3 Université Paris Saclay, Université Paris-Sud, Faculté de médecine, Le Kremlin Bicêtre, France; 4 Inserm Unit U981, Gustave Roussy, Villejuif, France; University Hospital of Navarra, SPAIN

## Abstract

**Introduction:**

Enrolling cancer patients in phase I clinical trials (P1s) requires that they fulfill specific criteria. Between the time they sign the consent form and the 1^st^ administration of the experimental drug, some patients may be excluded and considered as screen failures (SFs). Our objective was to assess SF patients profiles and the reasons and risk factors for SFs.

**Materials and Methods:**

All patients included in P1s at Gustave Roussy from 2008 to 2013 were reviewed retrospectively. SFs were matched with control P1 patients who were successfully enrolled. Patient and tumor characteristics, P1 types and the reasons for SF were analyzed.

**Results:**

Among 1,293 patients, 192 (15%) were SF cases; 182 SF cases were matched with 182 controls: median age was 57 (48–64) and 55 (47–63), median home-cancer center distance was 69 vs 55 km, 45% vs 34% had more than 2 metastatic sites, median screening period was 14 vs 11 days, median progression-free survival during the previous line was 12 vs 14 weeks, 37% vs 29% of LDH values were above the upper limit of normal, 42% vs 36% of albumin values were < 35 g/L, respectively. Reasons for SFs were cancer progression (44%), sponsor decision unrelated to a clinical reason (25%), patient retrieval (13.5%), relevant comorbidity (13.5%). Multivariate analysis revealed that a high Royal Marsden Hospital (RMH) prognostic score was potentially associated with higher risk of SFs (OR = 2.3; 95% CI [1.0–5.7], p = 0.06).

**Conclusion:**

Cancer progression led to half of the SFs in P1s. Physicians should pay attention to the RMH score at the time of patient inclusion to avoid further SFs.

## Introduction

There is an urge to develop new anticancer drugs in order to improve patients’ outcome. Therefore, the drug development process has to be quick and efficient, so that active drugs can be approved and made available to the largest number of patients as rapidly as possible. This process requires testing these drugs through successive phases of clinical trials, and obtaining approval takes several years [1]. Phase 1 clinical trials (P1s) are the first step of drug development [2], and often the first evaluation of such drug in humans (first-in-man or first-in-class trials). For obvious safety reasons, the patient’s health conditions must match protocol-specified “eligibility criteria” to enable patients to be enrolled [3]. The screening process takes place between the time the patient signs the informed consent form—which is the time of inclusion in a P1 and the very first administration of the experimental drug (cycle 1 day 1, C1D1). However, the health condition of previously included patients may evolve during the screening period. These patients may no longer fulfill inclusion criteria at the time of C1D1, and thus become screen failures (SFs). The occurrence of SFs decreases patient accrual onto P1 clinical trials, may make other patients miss a precious slot, is uselessly costly as it prolongs the experimental drug development process and thereby the time to drug approval. Finally, a SF is a time-consuming issue both for patients and physicians, as well as a financial burden for pharmaceutical companies [4].

The objective of our study was to determine the clinical and demographic criteria associated with the occurrence of SFs among patient candidates for P1 clinical trials, as well as the reasons for SFs. A retrospective case-control analysis aimed at assessing the related risk factors.

## Materials and Methods

### Study design and population

This case-control study was conducted using the clinical database of the Drug Development Department, Gustave Roussy Cancer Campus, Villejuif. The Ethics Committee of Gustave Roussy Cancer Campus approved this retrospective study. Patient records were anonymized and de-identified prior to analysis, as consent could not be obtained. All patients who were referred to the department medical team and who were included in any P1 clinical trial from 2008 to 2013 were eligible. Cancer patients who were considered as fully eligible during the enrolment visit but who were no longer eligible at the time of C1D1, and therefore never received the experimental drug, were considered as SFs. SFs due to the absence of the protocol-stipulated molecular aberration were excluded from the study. Each patient in the control group was blindly matched with a patient in the SF group for the P1 type.

### Reasons for screen failures

The reasons for P1 SFs were assessed and classified into four groups: (1) Withdrawal of consent and potential non-compliance were considered as a lack of personal involvement on the part of the patient; (2) Sponsor-induced SFs, including: sponsor’s decision to stop inclusion, unavailability of a slot for experimental drug administration, incompatibility of the imaging technique with tumor evaluation criteria (for example, the protocol stipulated CT scanning, whereas metastases are only visible on bone scan or on FDG-PET), asymptomatic complementary investigations not included in criteria (inadequate lab values and electrocardiogram abnormalities); (3) clinical cancer progression (decrease in the Eastern Cooperative Oncology Group Performance Status (ECOG PS), discovery of previously unknown brain metastasis); (4) other new relevant comorbidities (physical examination abnormalities, prohibited concomitant medication or any relevant interval medical issue).

### Data collection

The electronic medical records were reviewed retrospectively by one single investigator. We collected relevant data at patient inclusion in P1. Demographic characteristics included patient gender, age, geographical distance between home and the Gustave Roussy Cancer Treatment Center, referral or not from outside the cancer center and the number of concomitant medications. Collected tumor-related characteristics were as follows: primary tumor site, number and type of metastatic sites, ECOG PS, number of previous chemotherapy lines, progression-free survival (PFS) during the last chemotherapy line. The Royal-Marsden Hospital (RMH) prognostic score was determined for each patient based on the clinical and biological data available when the patient signed the informed consent form (ICF). This prospectively-validated score divided patients into two prognostic groups according to the albumin level (<35 versus ≥35 g/L), lactate dehydrogenase (LDH) values (concentration ≤ or > upper limit of normal (ULN)), and the number of metastatic sites (≤ or > 2) [5]. Finally, P1 characteristics were collected: wash-out duration, i.e. the time between the last administration of any anticancer drug and the forthcoming P1 C1D1; time between patient inclusion in P1 and C1D1; type of P1 experimental drug (intracellular signaling pathway inhibitors of tyrosine kinase domains, angiogenesis inhibitors, cytotoxics, immunotherapy, and hormone therapy), the patient’s previous inclusion and a further offer to participate in other P1s.

### Statistical analysis

The proportions of distinct reasons for SFs, with their 95% confidence intervals (95%CI), were computed in the case population, using a binomial approximation. Patient characteristics were described as N (%) for qualitative variables and medians [Q1-Q3] for quantitative variables. A multivariate conditional logistic regression on matched data was performed between both case and control populations. The statistical association between clinically-presumed SF risk factors and the occurrence of a SF was characterized by an odds ratio (OR) and the 95%CI. We made the hypothesis that 4 clinically-relevant covariates could be associated with the outcome independently of one another. The independent respective effect of RMH score (high versus low), time between signing the P1 informed consent form and C1D1 (<14 versus ≥ 14 days), duration of PFS during the previous line (≥ 10 versus < 10 weeks) and distance between home and cancer center (<100 versus ≥100 km) on occurrence of screening failure were derived from the conditional multivariable logistic regression for case-control pairs matched on P1 type. All tests were two-tailed and *p* values were estimated. Data were analyzed using SAS Software v9.2, (SAS Institute Inc. Cary NC, USA).

## Results

### Patient characteristics

From January 2008 to December 2013, 1,293 patients were included into P1s in the Drug Development Department, Gustave Roussy Cancer Treatment Center, Villejuif. Among them, 192 (15%) patients were identified as SF; 182 SF cases and matched with 182 controls. The main characteristics of the patients are summarized in Table 1. Fifty-eight percent of cases versus (vs) 46% of controls were referred from outside the Gustave Roussy center and 49% vs 43% lived further than 100 kilometers from the Gustave Roussy center, respectively. Lung cancer was the most frequent primary tumor site in both groups (19%); 45% vs 34% of patients had more than two metastatic sites; 37% vs 29% of LDH values were above the upper normal limit; 42% vs 36% of albumin levels were < 35 g/L, and the duration of PFS after the previous chemotherapy line was 12 ([9–18]) vs 14 weeks ([10–30]), respectively. The median time between inclusion and C1D1 was 14 days ([9–22]) in the study group and 11 ([7–16]) in the control group. Table 2 summarizes the types of phase 1 clinical trials in which the same proportion of both case and control patients were included.

**Table 1:** Patient characteristics at the time of signing the informed consent form (inclusion).

Variables	182 Cases		182 Controls
	n (%) or Median [Q1-Q3]		
**Demographic characteristics**			
Male	107 (59)		98 (54)
Age, yrs	57 [48–64]		55 [47–63]
Referred from outside the cancer center	105 (58)		83 (46)
ECOG PS 0	59 (32)		85 (47)
1	112 (62)	93 (51)	
Distance home-cancer center (km)	69 [20–397]		55 [19–241]
Number of medications	3 [2–5]		2 [0–3]
**Tumor characteristics**			
Primary tumor site			
lung	34 (19)		34 (19)
colorectal	23 (13)		27 (16)
melanoma	18 (10)		16 (9)
breast	16 (9)		18 (11)
prostate	15 (8)		9 (5)
Metastatic site			
liver	80 (44)		74 (41)
brain	27 (15)		3 (2)
bone	62 (34)		38 (21)
peritoneum	21 (12)		9 (5)
Number of previous lines	3 [2–4]		3 [1–4]
Duration of PFS after previous line (weeks)	12 [9–18]		14 [10–30]
Poor prognostic RMH score (≥2)	75 (41)		58 (32)
**Phase 1 clinical trial characteristics**			
Wash-out (days)	56 [36–85]		56 [35–164]
Time between inclusion and C1D1 (days)	14 [9–22]		11 [7–16]
Previous inclusion in a P1 trial	23 (13)		19 (10)
Further offer to participate in a P1 trial	17 (9)		52 (29)

Abbreviations: ECOG PS, Eastern Cooperative Oncology Group Performance Status; P1, phase 1; RMH, Royal-Marsden Hospital; C1D1, Cycle 1 day 1.

**Table 2:** Types of phase 1 clinical trials in which cases and controls were included.

Variables	n (%)
Intracellular signaling pathway inhibitors	77 (43)
Angiogenesis inhibitors	35 (19)
Cytotoxics	28 (15)
Immunotherapy	24 (13)
Hormonal therapy	9 (5)
Other*	9 (5)

* Other refers to trials of: HDAC inhibitors, cell adhesion inhibitors.

### Reasons and risk factors for SFs

Table 3 summarizes the reasons for SFs. In 46% of cases, cancer progression led to SFs. Protocol-specified non-clinically relevant criteria or a sponsor’s decision was involved in up to a fourth of SFs. Conversely, 13.5% of SFs were due to the absence of personal patient involvement; and a similar proportion of SFs occurred because of clinically relevant comorbidities. In the multivariate analysis, a poor Royal Marsden Hospital (RMH) prognostic score was associated with SFs (OR = 2.3; 95%CI [1.0–5.7], p = 0.06), although this was of borderline significance likely because of small numbers. Other factors included in the multivariate analysis such as PFS during the last chemotherapy line before the P1 experimental drug over 10 weeks (OR = 0.9; [95%CI: 0.4–1.9], p = 0.8), the time between signing the P1 informed consent form and C1D1 over 14 days (OR = 1.3; 95%CI [0.6–2.9], p = 0.6), and the distance between the patient’s home and the Gustave Roussy Cancer Treatment Center over 100 km (OR = 1.2; 95%CI [0.5–2.7], p = 0.7) were not associated with SFs.

**Table 3:** Reasons for screen failures in matched cases.

Variables	n (%)
**Clinical cancer progression**	**84 (46)**
Increased ECOG PS	54 (30)
Discovery of brain metastases	26 (14)
Discovery of other distant metastases	4 (2)
**Sponsor**	**46 (25)**
Inadequate lab values	30 (16)
Imaging not compatible with tumor evaluation	6 (3)
ECG abnormalities	5 (3)
Sponsor decision or slot not available	4 (2)
Concomitant medications	1 (<1)
**No personal involvement on the part of the patient**	**24 (13.5)**
Consent withdrawal	23 (13)
Potential patient non-compliance	1 (<1)
**Relevant comorbidities**	**20 (11.5)**
Physical examination abnormalities*	15 (8)
Interval medical issue	5 (3)
Other	8 (4)

Abbreviations: ECG, electrocardiogram; ECOG PS, Eastern Cooperative Oncology Group Performance Status; P1, phase 1.

* Physical abnormalities include 7 cases of decreased left ventricular ejection fraction, 4 cases of pulmonary function test abnormalities, 2 cases of infection, 1 case of renal failure, 1 case of neuropathy

## Discussion

One in every six patients included in a P1 in our department was likely to be considered as a screen failure, which signifies exclusion from the trial before any experimental drug administration. Cancer progression was responsible for nearly half of the cases which may have been related to a high (>2) RMH prognostic score which is synonymous with a poor prognosis.

Such a proportion of SFs in P1s is consistent with that found in the published literature. In a monocentric retrospective study performed among 773 American cancer patients included in P1 trials, a quarter of them were SFs [6]. We considered a more restrictive definition of SFs, only focusing on avoidable cases. We excluded 52 cases that McKane *et al*. might have considered in their report, like SFs due to the absence of a biomarker required by the study protocol. Our P1 department clinicians perform a very thorough selection of potentially-eligible patients, based on their medical history and a blood test performed within the two previous weeks. Thus, 70% of patients referred to our unit are discarded before signing any P1 ICF. The most common reasons for SFs were similar. Out of range lab results led to a 26% rate of SFs, versus 16% in our study. The withdrawal of patient consent was the reason for a SF in about 13.5% of cases. Our study emphasizes how restrictive sponsor-specified inclusion criteria may be in clinical trials [7]. Indeed, up to a quarter of SFs were related to non-clinically significant criteria or P1 feasibility, and these reasons led to 38% of SFs in the series of Gerber et al. In parallel, 14% of SFs were related to the discovery of brain metastasis in our study. The clinical relevance of such exclusion criteria is still under debate in the P1 field [8,9]. The positive clinical outcomes obtained in highly selected patients enrolled in clinical trials might therefore be an overestimation of the supposed clinical benefits to patients in the “real-life” care context [10]. Most importantly, almost half of our SF cases were due to cancer progression. This highlights the importance of precisely assessing patient’s fitness when they sign the ICF. RMH score might be part of a broader set of clinical and biological criteria to help physicians to better select patients more likely to remain eligible (according to protocol-specified inclusion criteria) at the time of C1D1. Further studies are warranted to assess effective tools to select properly patients before ICF signature.

Few published data are available on our study topic and the definition of a SF is not homogeneous in the literature [11]. Besides describing the profiles of 182 SF patients, our analysis provided the reasons for SFs in P1s. Matching our cases with controls enabled us to identify factors associated with such an issue. However, retrospective data collection may have induced evaluation bias and excluded relevant missing data, such as the duration of patient survival after a SF.

Avoiding the occurrence of SFs is an ethical issue. First, SFs may decrease the absolute number of patients receiving P1 experimental drugs. Yet, including patients in P1s allows them to gain access to currently unapproved anticancer drugs, which might provide them with significant clinical benefits, such as enhanced overall survival (OS) and/or quality of life. Second, the occurrence of SFs may delay the administration of next-generation anticancer drugs to patients with progressive cancer. Patient health conditions may deteriorate due to a prolonged period without anticancer treatment. For example, in our study, a new P1 inclusion was offered to one third of controls but only to 9% of SF patients.

By paying particular attention to the RMH score when the patient signs the P1 informed consent form, physicians may avoid early exclusions, mostly due to cancer progression, of one in every six patients potentially eligible for P1. This is of key importance, first in order to avoid offering to the patient unethical hope of a treatment that will eventually never start, but also in order to optimize the drug development process and efficiently bring active drugs to registration.

## References

[1] DiMasi JA, Grabowski HG. Economics of new oncology drug development. J Clin Oncol. 2007;25: 209–16. 17210942 10.1200/JCO.2006.09.0803

[2] Ivy SP, Siu LL, Garrett-Mayer E, Rubinstein L. Approaches to phase 1 clinical trial design focused on safety, efficiency, and selected patient populations: a report from the clinical trial design task force of the national cancer institute investigational drug steering committee. Clin Cancer Res. 2010;16: 1726–36. 10.1158/1078-0432.CCR-09-1961 20215542 PMC5207802

[3] Fu S, McQuinn L, Naing A, Wheler JJ, Janku F, Falchook GS, et al Barriers to study enrollment in patients with advanced cancer referred to a phase I clinical trials unit. Oncologist. 2013;18: 1315–20. 10.1634/theoncologist.2013-0202 24153239 PMC3868426

[4] Blenkowski RS, Goldfarb NM. Screen failures in clinical trials: financial Roulette or the cost of doing business? J Clin Res Best Pract. 2008;4: 1–4.24526930

[5] Arkenau HT, Barriuso J, Olmos D, Ang JE, de Bono J, Judson I, et al Prospective validation of a prognostic score to improve patient selection for oncology phase I trials. J Clin Oncol. 2009;27: 2692–6. 10.1200/JCO.2008.19.5081 19332724

[6] Mckane A, Sima C, Ramanathan RK, Jameson G, Mast C, White E, et al Determinants of patient screen failures in Phase 1 clinical trials. Invest New Drugs. 2013;31: 774–9. 10.1007/s10637-012-9894-7 23135779

[7] Gerber DE, Pruitt SL, Halm EA. Should criteria for inclusion in cancer clinical trials be expanded? J Comp Eff Res. 2015;4: 289–91. 10.2217/cer.15.27 26274789 PMC4838010

[8] Carden CP, Agarwal R, Saran F, Judson IR. Eligibility of patients with brain metastases for phase I trials: time for a rethink? Lancet Oncol. 2008;9: 1012–7. 10.1016/S1470-2045(08)70257-2 19071257

[9] Hollebecque A, Postel-Vinay S, Verweij J, Demetri GD, Flaherty K, Bedard P, et al Modifying phase I methodology to facilitate enrolment of molecularly selected patients. Eur J Cancer. 2013;49: 1515–20. 10.1016/j.ejca.2012.12.012 23333057

[10] Unger JM, Barlow WE, Martin DP, Ramsey SD, Leblanc M, Etzioni R, et al Comparison of survival outcomes among cancer patients treated in and out of clinical trials. J Natl Cancer Inst. 2014;106: dju002 10.1093/jnci/dju002 24627276 PMC3982777

[11] Wang D, Pearce T, Cobani V, Zekaj M, Adams N, Williamson A, et al Lessons from the other side of clinical trial accrual: Screen failures at the Josephine Ford Cancer Center/Henry Ford Health System in 2010. J Clin Oncol 29:Abstr 16624.

